# Influence of ergonomic layout of musician chairs on posture and seat pressure in musicians of different playing levels

**DOI:** 10.1371/journal.pone.0208758

**Published:** 2018-12-11

**Authors:** Daniela Ohlendorf, Christian Maurer, Elisabeth Bolender, Veronica Kocis, Martha Song, David A. Groneberg

**Affiliations:** 1 Institute of Occupational Medicine, Social Medicine and Environmental Medicine, Goethe-University Frankfurt am Main, Frankfurt am Main, Germany; 2 Move-functional, Berndorf bei Salzburg, Austria; Hochschule Trier, GERMANY

## Abstract

**Introduction:**

Musicians often perform in forced postures over a long period of time, which in the worst case may lead to playing-related musculoskeletal disorders. In this context, the ergonomics of the musician's chair (construction and surface quality) can be an influencing factor, with impact on the seating position of the upper body and the pressure distribution of the bottom. Therefore, the relationship between different musician chairs and musicians of different playing levels (professional, amateur or student) was analyzed in order to gain useful insights whether playing experience, playing level, playing style (symmetrical or asymmetrical) or gender have an impact.

**Method:**

The total dataset of 47 musicians (3 playing levels: professional, amateur, student) were analysed on six musician chairs with different ergonomic layout. Sitting on each chair without instrument (condition 1) and with instrument (condition 2), the upper body posture (videorasterstereography) and the seat pressure (load distribution) were recorded.as Also, a subjective assessment concerning constitutional data, sitting behaviour, prevailing pain in the musculoskeletal system, sport activity and chair comfort rating, was completed using a questionnaire.

**Results:**

There were significant differences shown in 6 of 17 variables, where all between and within factors were accounted for with a MANOVA. Two measurements of the upper body posture (scapular distance and scapular height) differentiated between playing level. Four of the pressure measurements (pressure under the sit bone and the thigh for the left and the right side) differentiated between chairs and the two conditions (with and without instrument). Chairs with soft cushioning had a mean pressure reduction of about 30%. The pressure was increased by about 10% while playing an instrument. Subjective rating was correlated to age for some of the chairs.

**Discussion:**

Differences between chairs are mainly associated with the pressure distribution under the sitting surface. Playing with an instrument puts an additional force onto the surface of the chair that is more than the weight of the instrument. No relationship between pressure data and upper body posture data could be found. Therefore, it can be speculated that the intersubject variability is larger than systematic differences introduced by the chair or instrument.

## Introduction

In the learning of a musical instrument, fast, repetitive, asymmetrical, complicated movements of the arms, hands and fingers are trained. In order to support these movements, the torso simultaneously performs static holding work. These combined movements of dynamic and static muscle work in the respective body segments accumulate with increasing performance level in relation to intensity, density, duration, extent, occurrence and frequency of movement. The resulting loads on the movement systems are related to the instrument-specific performance, and in the worst case can cause overloads. These can lead to symptoms such as pain in the chin, back, neck, shoulder, arms and hands [1]. These symptoms are also associated with playing-related musculoskeletal disorders (PRMD) [2]. These disorders appear in professional musicians [3–7] and music students [8–10], as well as in amateur orchestra musicians [11, 12], and are a central theme of complaint. These PRMD are generally documented in many questionnaires [7, 9, 10, 13–19], whereas women have a higher prevalence [7, 17, 20]. From previous studies, it can be concluded that taking the typical playing position causes unfavourable postures. Furthermore, low physical compensation possibilities while playing constitute a high predictive factor for physical complaints in the musculoskeletal system. The effects of music making on different regions of the musculoskeletal system have so far been recorded in many cases using different measuring techniques [21–28]. In addition to these personal characteristics when playing music, which are often very difficult to change, it is also possible to remedy the situation through a change of external conditions. According to Vervainioti and Alexopoulos [29] it is not only the musician-specific posture that can be identified as a job-related stressor. They also point out, factors such as public exposure, personal hazards, repertoire, competition, job context, injury/illness, and criticism should not be neglected. Kok et al. [20] recommend focusing on associated risk factors to reduce PRMD, due to the fact that there is little scope for movement variation in posture when playing. Such associated risk factors can either be the repertoire, such as technical demands or environmental conditions. Environmental conditions include the chair on which the musician sits while playing music. In this context, the ergonomics of the musician's chair—the construction of the chair, as well as the surface quality of the seat—is a factor to be taken into account. These components are very important, because a musician often has to stay in a certain playing position over a long period of time. Accordingly, an "ideal" ergonomic chair, which corresponds to the physical demands of a musician, is a meaningful way of counteracting or reducing PRMD.

In order to be able to quantify the different ergonomic concepts of the chairs with regard to musical posture, the distribution of sitting pressure is just as important as the upper body posture, which must be assumed to adapt cranially to the pelvic position during sitting. Whether different music chair concepts depending on the playing position in comparison to the usual sitting posture have varying effects on these aspects is the subject of this study. In order to gain insight into whether the playing experience, the playing level (professional, amateur or student), the playing style (symmetrical and asymmetrical way of playing) or the gender selection criteria are similarly affected by sitting position, a broad spectrum of musicians was selected for participation in this study. The following hypotheses are to be tested:

Hypothesis 1: Chair geometry has an influence on the sitting position.

Hypothesis 2: Sitting position depends on the playing level of musicians.

## Material und methods

### Subjects

A total of 64 (25f/39m) musicians were measured for this study. Among them were 20 (9f/11m) music students, 21 (14f/7m) amateur musicians with at least 8 years of praxis in general and 23 (2f/21m) professional musicians aged between 18 to 60 years (33.6 ± 12.7 years). Since only complete data sets were used for data analysis, 17 musicians had to be excluded. This results in a group of 47 right-handed test persons (32.4 ± 13.2 years) who were recruited for this study. Experience level ranged from 20 (13f/7m) amateur musicians, over 10 (7f/3m) music students at university level to 17 (2f/15m) professionals playing in an orchestra. Subjects played different instruments that could be classified into two groups: symmetric instruments, such as clarinet, trumpet and saxophone, and asymmetric instruments, such as violin, guitar and concert flute. All characteristics of the participants are additionally listed in Table 1.

**Table 1:** Subjects characteristics.

Variable	Amateurs	Students	Professionals
Age	24.50 ± 24.47	23.80 ± 13.51	46.76 ± 10.36
Gender	13f/7m	7f/3m	2f/15m
Mass [kg]	65.00 ± 10.08	60.20 ± 20.04	82.94 ± 13.28
Height [cm]	176.05 ± 7.69	165.00 ± 41.06	179.82 ± 8.00
BMI [kg/m^2^]	20.85 ± 2.01	21.87 ± 5.85	25.89 ± 3.59
Played instrument	Symmetrical: 9 Clarinet, Horn, Keyboard, Trumpet, Bassoon Asymmetrical: 11 guitar, violin, flute	Symmetrical: 3 Fagott, Cello Asymmetrical: 7 Violin	Symmetrical: 15 Tuba, Trombone, Horn Trumpet Drum, Saxophone, Double Bass, Bassoon, Clarinet Asymmetrical: 2 Flute

Exclusion criteria were traumatic lesions, such as fractures, herniated discs or torn ligaments, undergoing surgery within the last six months, taking analgesics or muscle relaxant medication or suffering from genetic muscular or neurological diseases.

All participants provided written permission to participate in this study. The study is approved by the ethics committee of the Medical Faculty of the Goethe-University (Nr. 14/16).

### Measurement systems

#### Back scan

The back scanner „MiniRot-Kombi”(ABW GmbH; Frickenhausen / Germany) scanned the upper body posture by video raster stereography. In the used standard mode, one surface recording delivers up to 307200 coordinates of the surface within 0,6 sec and with an approximate resolution of 1 mm in the object plane (depth). The surface coordinates are mapped in a 3D Cartesian coordinate system because of the curvature of the surface. In the used standard mode recording was done with 50Hz frame rate. Depth resolution was 0.01 mm. Resolution was improved with six skin markers and repeated measurements done below 0.5 mm [30]. Several measurement variables can be calculated from the surface measurement. A selection of 9 relevant variables was used in this study: frontal and sagittal trunk decline (FTD and STD), axis decline (AD), scapular distance (SD), scapular height (SH), scapular rotation (SR), pelvic height (PH), pelvic rotation (PR)and pelvic torsion (PT). A detailed description of the parameters can be found in [30].

#### Pressure measuring sitting mat

The pressure onto the sitting surface was measured with the GP SoftMess sitting mat (GeBioM, Münster, Germany). The pressure mat has a size of 54cm x 57cm with an active area of 48cm x 51cm. A total number of 256 resistive sensors are equally distributed; therefore each sensor covers an area of 2 cm^2^ [31]. Again, measurements were recorded for one second with a sampling rate of 30Hz. The measurement range was 0.02 to 0.5 N/cm^2^ with a precision of 5%. Seven variables were used in this study: pressure left to right [%] (PLR), point of load incidence in transversal axis [mm] (POL), loaded area [mm^**2**^] (LA), mean pressure sit bone left and right [mbar] (mPSL and mPSR), mean pressure thigh left and right [mbar] (mPTL and mPTR).

### Chairs for musicians

**Fig 1:**
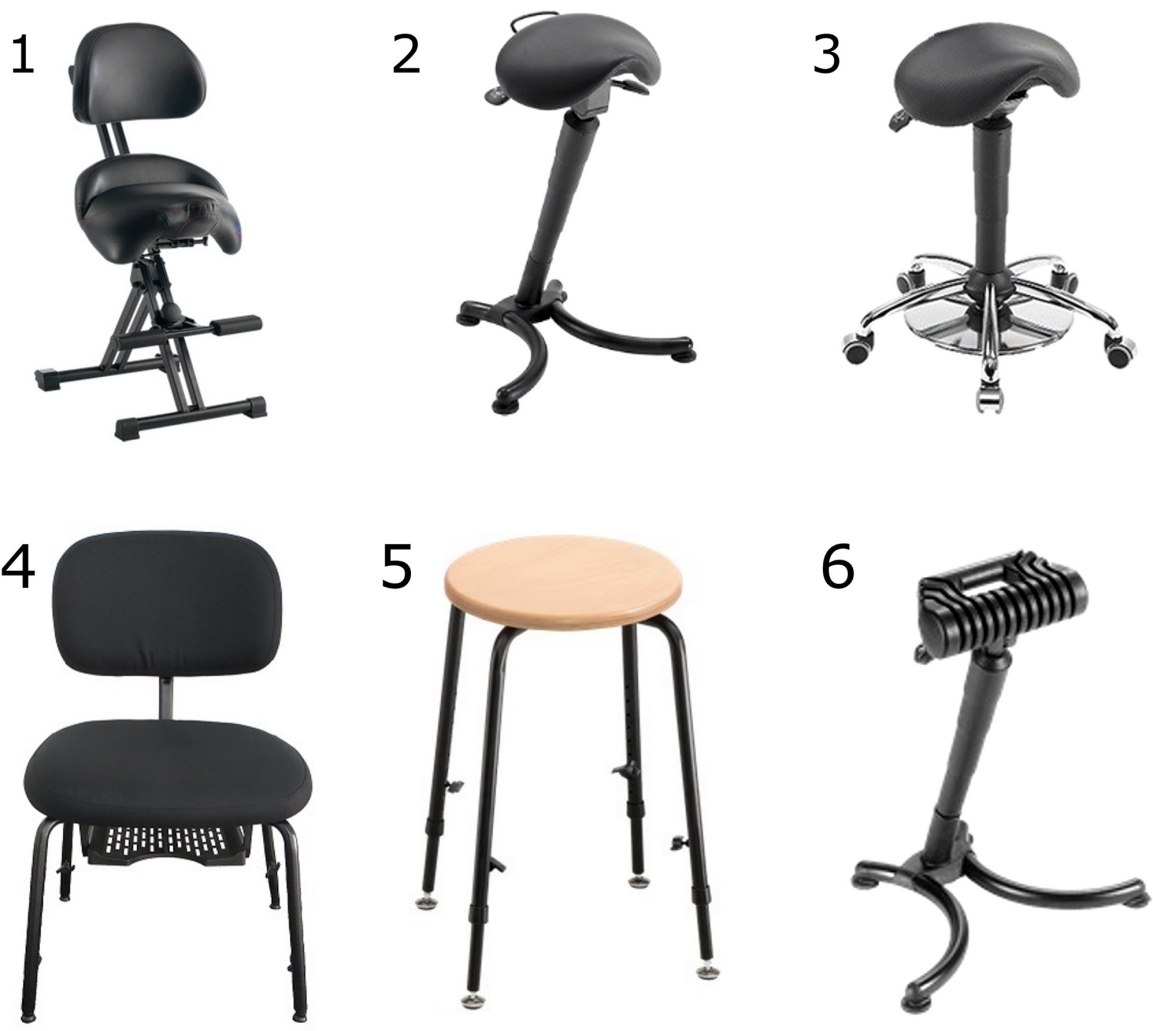
Chairs used. 6 different ergonomic music chairs, used by professional musicians are examined.

Six chairs constructed for musicians from the company Mey Chair Systems (Sesslach-Merlach, Germany) were used. The chairs cover a wide range of commercially available constructions and are widely used by amateur and professional musicians. The chairs follow different ergonomic principals and vary in the geometry and texture of the sitting area (Fig 1). The main characteristics of the chairs are listed in Table 2.

**Table 2:** Chair characteristics. List of all six chairs including: special attributes, sitting area and height of sitting position.

Chair	Special attributes	Sitting area	Height of sitting position (knee angle)
Chair 1	- padded backrest - Sitting height and inclination of the backrest not adjustable	Triangular/saddle (width of 35cm)	Not adjustable: 73cm
Chair 2	- No backrest	Triangular/saddle with a width of 35cm Able to bend forward and backward	66 cm– 87 cm (knee angle: up to 120°)
Chair 3	- No backrest		56 cm– 70 cm (knee angle: approx. to 90°)
Chair 4	- Backrest removed	cushioned	41 cm– 56 cm
Chair 5	- No backrest	Wooden (diameter of 34cm)	45 cm– 65 cm
Chair 6	- No backrest	Ergonomically formed	63cm– 84 cm

### Subjective assessment

Further statistical material was gathered through a questionnaire. Questions included constitutional data (age, sex, body height, body weight, handedness), sitting behaviour (sitting hours per working day), an assessment of prevailing pain in the musculoskeletal system, sport activity as well as the comfort rating of all musician chairs. The questions were to be answered on a five-step scale, whereby the sitting behaviour has gradations of 20% steps, sport activity is rated in hours/week (<1h to>7h) and complaints of the musculoskeletal system are differentiated in various individual body regions. The assessment of chair comfort is based on the school grading system, with 1 representing the best and 6 the worst rating.

### Measurement protocol

**Fig 2:**
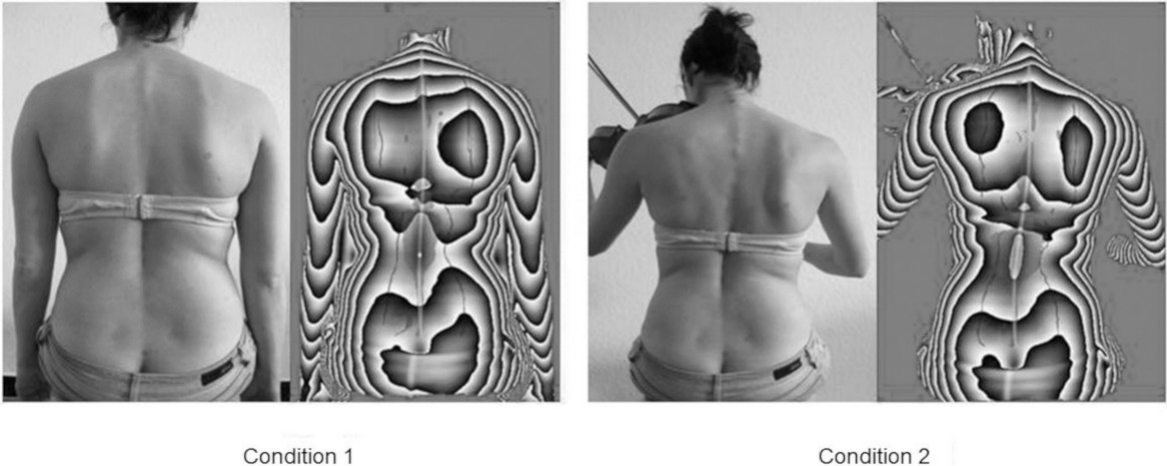
Photo and phase image of both conditions. The left side shows the condition without instrument (condition 1) and the right side with instrument (condition 2).

Six markers were placed on landmarks on the bare skin of the back. These markers were used to improve the accuracy of the back scan. Subjects had to sit with and without an instrument on the chair (condition, Fig 2). For both conditions subjects were instructed for an ergonomic sitting position. The ergonomic sitting position was based on the recommendation of the manufacturer. Sitting position differed slightly between the various instruments, as differing postures are required in order to perform best on each instrument. For the demonstration of the correct sitting position, a goniometer was used. Subjects were instructed to look at the music stand that was placed in front of them. The measurements of the back scan and the pressure distribution of the sitting area were done simultaneously. Every subject was measured at least three times, sitting on each chair. For statistical analysis, the mean value of the three measurements was used. The chairs were used in randomised order. The back scan could not be measured on the 1^st^ chair, and the bended hard surface of the 6^th^ chair did not allow a measurement of the pressure distribution.

### Statistics

For the statistical analysis, several influencing factors were controlled. Therefore, we used a MANOVA model in order to account for multi comparison. Outliners (defined as values 5 standard deviations away from the variable mean) were removed from the data set by removing the whole subject from the data set. Forty-seven subjects remained with good and complete data sets. The mutual independence of the variable was checked with a correlation analysis. The cut on for dependence was set to r = 0.75. Prior to the MANOVA the relationship between the two measurement systems was checked. This was done using a factor analysis with a varying number of factors (between 3 and 8).

All mutual independent variables were controlled for normal distribution (Lilifort test). As variables were not normally distributed, data were transformed to normal distribution by applying an inverse normal distribution to the ranks of the data. The MANOVA was set up with 3 within factors and 6 between factors. Within factors were: the chair condition, the instrument vs. not instrument condition, as well as the variables of the back scan and the pressure sitting mat (for detailed description see the subsection measurement systems). Between factors were sex, age, weight, height, the playing level and the group of instrument (symmetric instrument vs. asymmetric instrument). Significant comparisons within the MANOVA were subjected to an ANOVA. For the following up ANOVA tests, the multi comparison correction was done by Tukey-Kramer correction. Also the subjective ratings were analysed within the factors chair, and condition, and between the factors of sex, age, weight, height, playing level and the group of instrument. Therefore, Friedman-Test followed by multiple Conover-comparisons was used. P-values were corrected by Bonferroni-Holm. Further, correlations between age and subjective evaluation of the chairs were calculated by using Spearman & Kendall rank correlation.

The force difference (ΔF) applied to the surface of the chair between the conditions with and without instrument was estimated with the formula:

ΔF = (P_w_ * A_w_−WI−P_wo_*A_wo_)* κ, where P_wo_ is the pressure without instrument, A_wo_ is the sitting surface without instruments, P_w_ is the pressure with instrument, A_w_ is the sitting surface with instruments, and W_I_ is the weight of the instrument. κ is a correction factor required because only the mean pressure was measured. If the force difference was greater than zero, more force was applied to the surface of the chair during playing an instrument.

The significance level was set to 0.05. All statistical analysis was done with matlab (mathworks, Version 2017b).

## Results

**Fig 3:**
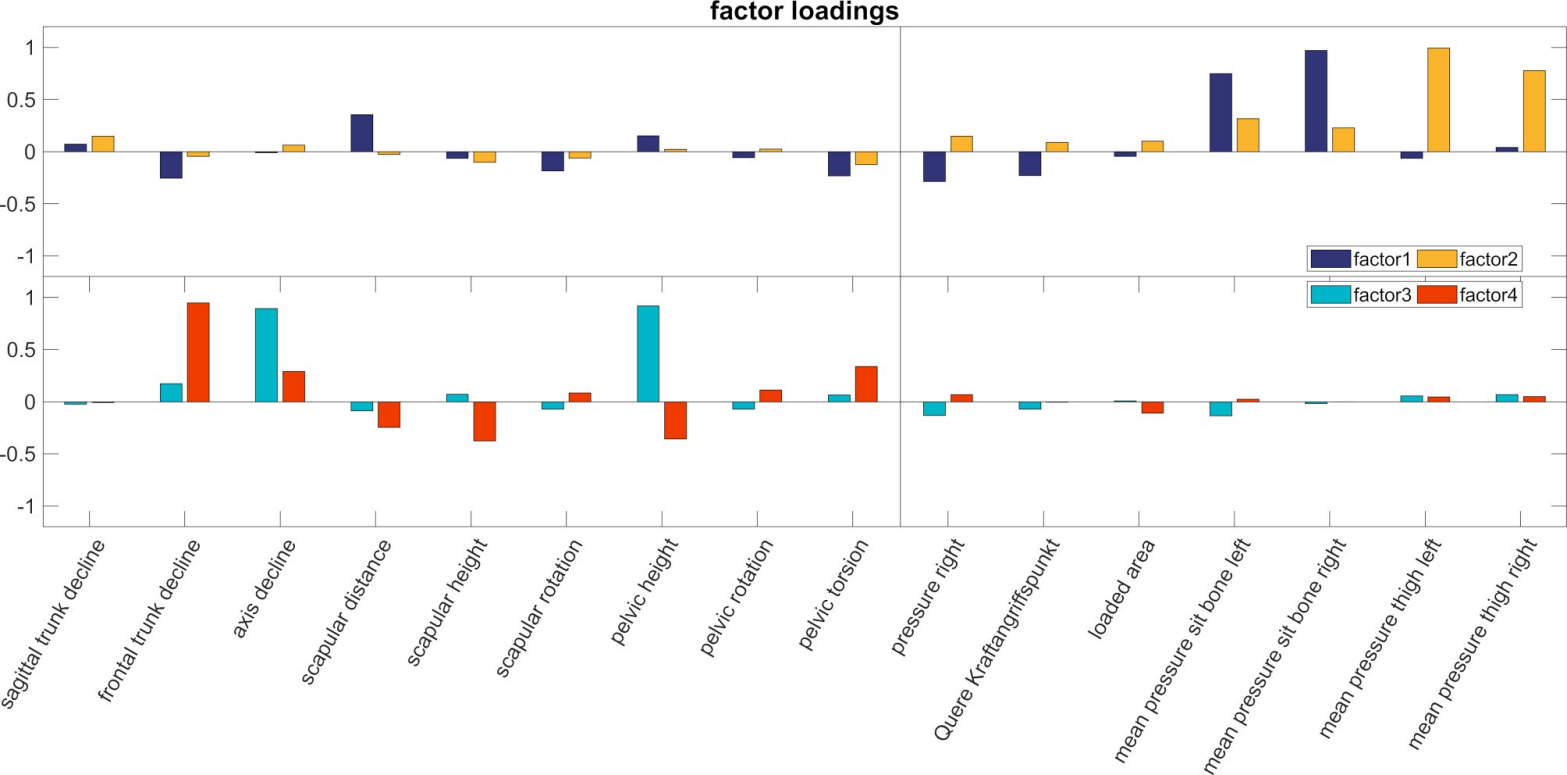
Factor loadings. The four factors that best describe the underlying data best are plotted. On the left side the data of the back scan are plotted. On the right side, the data of the pressure measurements are plotted. The first two factors (upper bar plot) load mainly on the pressure data, the 3^rd^ and 4^th^ factors load mainly on the back scan data.

Four factors achieved the optimal result when all measurement variables were combined (Fig 3). The two measurement systems load on different factors. The first two factors load mainly on pressure measurements, the 3^rd^ and 4^th^ factors load mainly on the back scan data.

Differences could be found between the interaction of level and weight within the sets of chair, condition and between the interaction of weight within the set of variables (Table 3). The subsequent post hoc tests revealed that six variables have significant differences.

**Table 3:** MANOVA table. Results of the underlying MANOVA model.

Within	Between	Statistic	Value	F	RSquare	df1	df2	p-Value
chair	symmetric instrument	Wilks	1.00	0.02	0.00	1	14	0.89
chair	sex	Wilks	0.98	0.26	0.02	1	14	0.62
chair	age	Wilks	0.81	3.26	0.19	1	14	0.09
**chair**	**weight**	**Wilks**	**0.56**	**10.96**	**0.44**	**1**	**14**	**0.01**
chair	height	Wilks	1.00	0.01	0.00	1	14	0.92
**chair**	**level**	**Wilks**	**0.75**	**4.74**	**0.25**	**1**	**14**	**0.05**
condition	symmetric instrument	Wilks	1.00	0.06	0.00	1	14	0.81
condition	sex	Wilks	0.99	0.19	0.01	1	14	0.67
condition	age	Wilks	0.78	3.95	0.22	1	14	0.07
**condition**	**weight**	**Wilks**	**0.47**	**15.65**	**0.53**	**1**	**14**	**0.001**
condition	height	Wilks	1.00	0.02	0.00	1	14	0.89
**condition**	**level**	**Wilks**	**0.69**	**6.17**	**0.31**	**1**	**14**	**0.03**
variable	symmetric instrument	Wilks	0.98	0.26	0.02	1	14	0.62
variable	sex	Wilks	0.98	0.36	0.02	1	14	0.56
variable	age	Wilks	0.79	3.83	0.21	1	14	0.07
**variable**	**weight**	**Wilks**	**0.42**	**19.04**	**0.58**	**1**	**14**	**0.001**
variable	height	Wilks	0.98	0.22	0.02	1	14	0.65
variable	level	Wilks	0.80	3.59	0.20	1	14	0.08

The interaction effect of level was mainly found within two variables of the back scan. These variables were the scapular distance and the scapular height. However, the three groups are different in age F(2,46) = 42 (p<0.001) with the students and amateurs being younger than the professionals, in weight F(2,46) = 13.8 (p < 0.001) with the students and the amateurs weight less than the professionals, in height F(2,46) = 9.5 (p<0.001) with the students being smaller than the amateurs and the professionals and in sex F(2,46) = 8.6 (p<0.001) with the professionals being mainly male. The group of the old, tall, heavier male professionals have a scapular distance of 197mm (± 23mm) and a scapular height of -3° (± 9°), while the group of the young, lightweight, tall, mixed gender students have a scapular distance of 175mm (24mm), and a scapular height of -7° (± 7°) and the group of the young, lightweight, tall, mixed gender amateurs having a scapular distance of 164mm (± 28mm) and a scapular height of -3° (± 8°).

**Fig 4:**
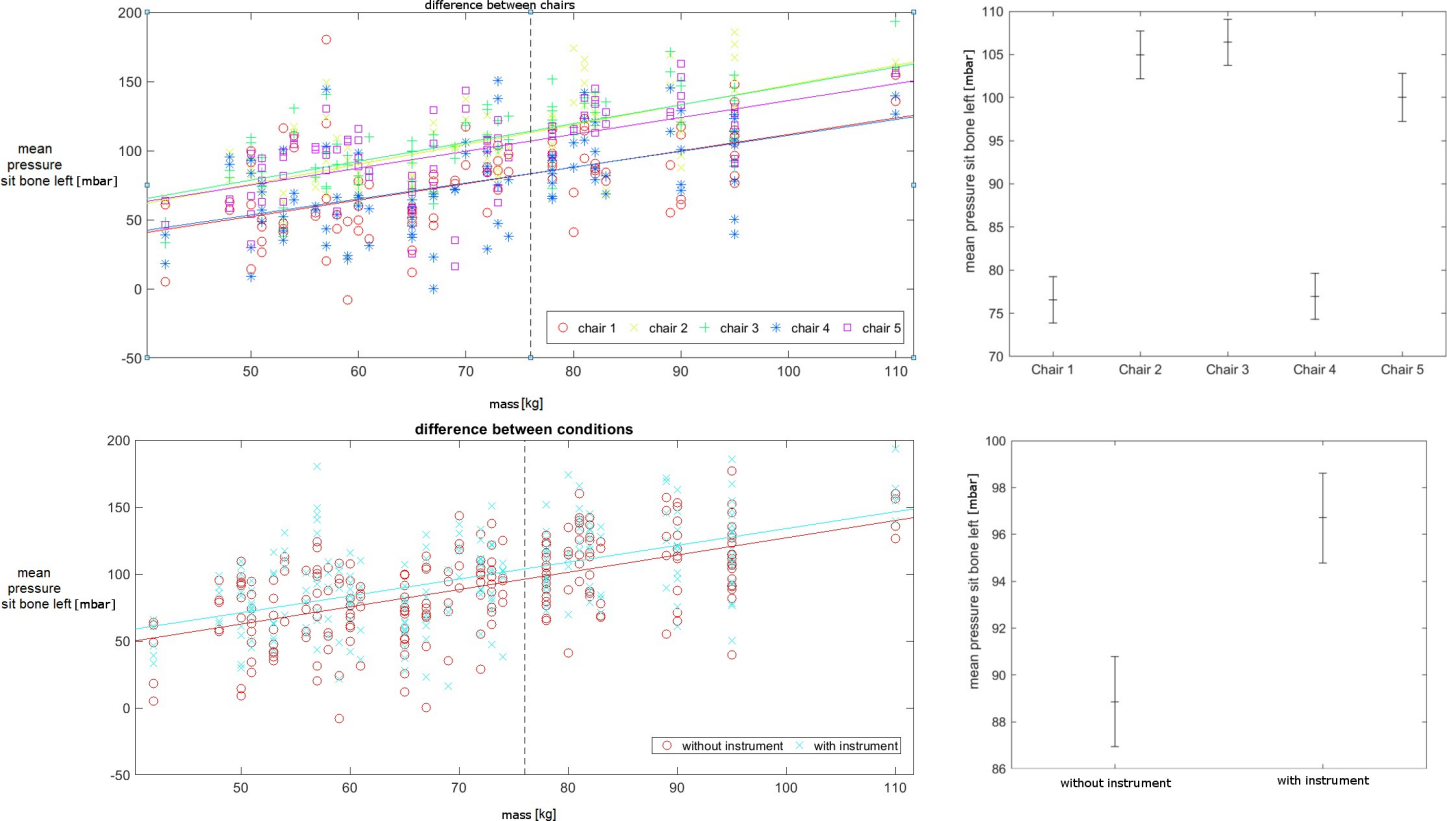
Differences between chairs and conditions. The upper row shows the comparisons for different chairs. The lower row shows the comparisons for the two conditions with and without instrument. The left side shows the plot with the covariance, and the right side shows the population marginal means.

The interaction effect of weight was mainly found within four variables of the pressure mat. These variables are the mean pressure under the tuber ischiadicum and under the thigh for the left and the right body side. The covariance was calculated for all four variables (Fig 4). There was no difference between the left and the right side. Therefore, Fig 4 shows only the results of the left side for the thigh and the tuber ischiadicum. For the mean pressure at the tuber ischiadicum, the difference between the chairs was F(4,443) = 31 (p<0.001) for the left body side and F(4,443) = 21 (p<0.001) for the right body side. For the thigh, the values are F(4,443) = 9 (p<0.001) for the left body side and F(4,443) = 8.3 (p<0.001) for the right side. Within the chairs, the first and the 4^th^ chair were similar and had lower pressure values than the other three chairs (chair 2, 3 and chair 5) (Fig 4, left side).

The two conditions (without and with instrument) are different for three of the four variables: The mean pressure at the tuber ischiadicum separates the two conditions by F(4,449) = 8.4 (p = 0.004) for the left body side and F(4,443) = 5.3 (p = 0.022) for the right body side. The mean pressure at the thigh is F(4,443) = 10 (p = 0.002) for the left body side and F(4,443) = 3 (p = 0.082) for the right body side (Fig 4, right side). There is no difference between the left and the right side. While playing an instrument, an additional pressure of 0.079N/cm^2^ was applied to the surface of the chair surface.

**Fig 5:**
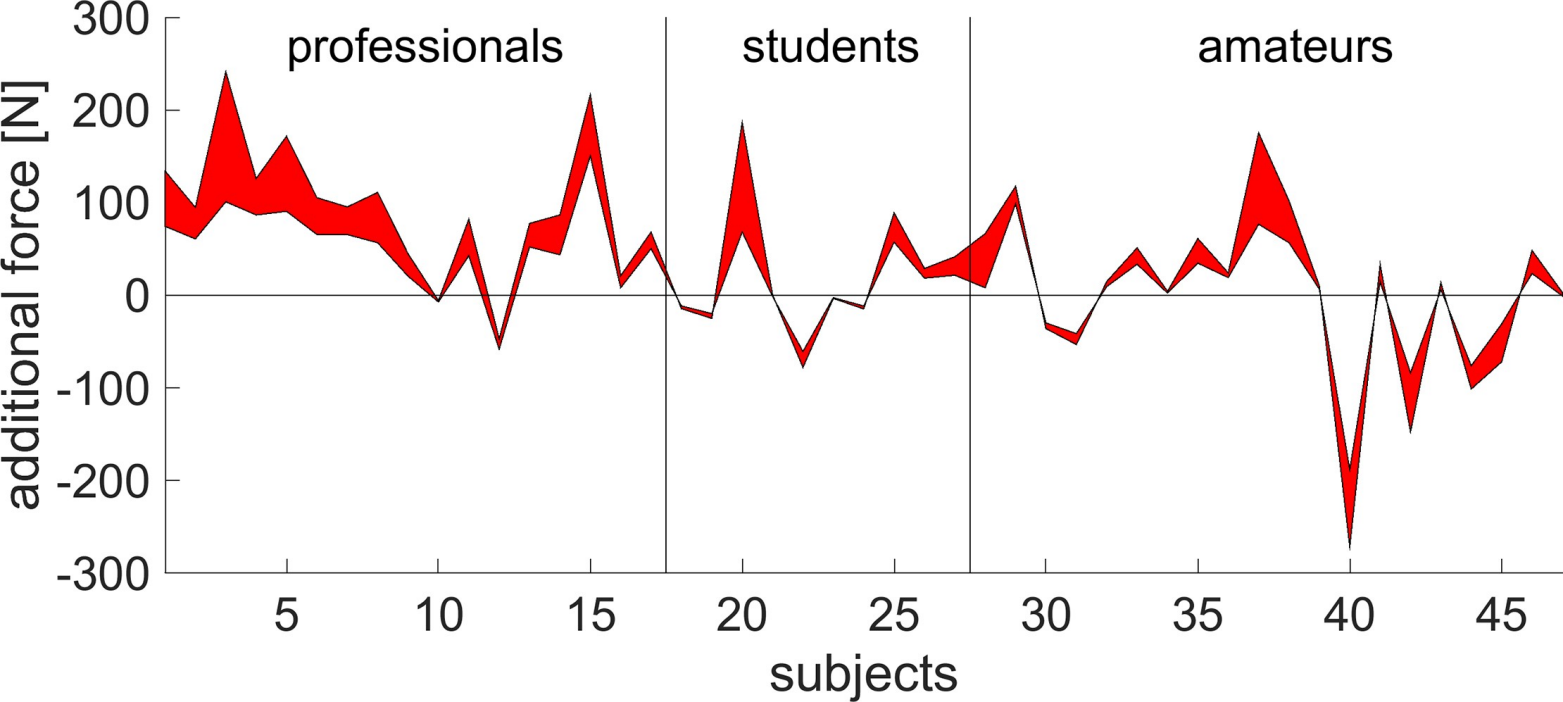
Additional force onto the surface of the chair while playing an instrument. For every subject, the confidence interval for the expected additional force was calculated.

The force difference applied to the surface of the chair was in general larger than zero (Fig 5). The range of the additional force is in the range of 52 N to 95 N mean of lower confidence interval to mean of upper confidence interval for the professional players. The additional force is from 5 N to 21 N mean of lower confidence interval to mean of upper confidence interval for the group of the students. For the amateur players the mean additional force is around zero, ranging from -3.4N to 2N. κ was estimated to be 4 ± 0.8.

Within the chairs, significant correlations can be found between subjective rating and age for chair 1, 2, 4 and 5 (Table 4). Effect sizes for these comparisons are at most weak. The chair 1, 4 and 5 are rated worse with increasing age, the chair number 2 is rated better with increasing age.

**Table 4:** Correlation of subjective rating. Correlations between age and subjective evaluation of the chairs. The p-values, the correlation coefficient (rho) and the effect strength according to Evans are shown. Significant p-values are marked in bold.

	p-value	rho	Effect size (Evans)
**Chair 1**	**0.01**	**0.36**	**weak**
**Chair 2**	**0.02**	**-0.31**	**weak**
**Chair 3**	0.81	0.03	poor
**Chair 4**	**0.01**	**0.32**	**weak**
**Chair 5**	**0.02**	**0.30**	**weak**
**Chair 6**	0.37	-0.12	poor

On a gender specific level, no difference can be found between the chairs for the male group. The female group shows a significant difference (p≤0.01) scoring between chair 5 and 6 (p≤0.03, rates 5 and 2, respectively). In addition, there is a negative correlation between hours of sporting activity per week and weight (p≤0.03; rho: -0.32 [weak]). No correlation could be found between age and sporting activity or between age and pain.

## Discussion

Within this study we have addressed the two main questions, whether (1) the chair geometry has an influence on the seating position, and if (2) the sitting position depends on the playing level of musicians. We therefore collected data of 47 musicians with three different playing levels. The collected data can be categorized in three groups, upper body posture data, pressure data and subjective assessment.

To answer the **first** hypothesis, the quantitative data were subjected to an overall analysis including all within and between factors. The first step, the factor analysis revealed that the two measurement systems are independent. Factors 1 and 2 loaded mainly on pressure data, while the factors 3 and 4 loaded mainly on upper body posture data (Fig 3). It has to be noted that the measurements are done within a more or less static position, in which the subjects placed their body in an individually optimal balanced position for their performance. There is also no difference between the measured values of the sitting position of the conditions “with instrument” and “without instrument”. It has to be noted that only the back of the upper body was measured, and not the arm position. While the arm position changes drastically while holding and playing an instrument, it seems that the back of the upper body does not change systematically. Reason for this can be that there is no difference in the two conditions, or more likely, that the variability between subjects is much larger than the systematic difference between these two conditions (Fig 3). Several studies have shown the larger intersubjective variability for the same movements [32–34]. However, the result of the factor analysis should not be interpreted that changing the body position does not change the pressure distribution. The relationship between body alignment and pressure is well understood by the laws of basic physics. The centre of pressure can be calculated from the weight and position of all segments. Within this study we have only shown, that there is no systematic relationship between specific pressure data and specific measures of the back in the static sitting position (Fig 3).

In the second analysis step, all quantitative data were subjected to a MANOVA (Table 1). This was done for the three within factors of chair, condition and variables, and the six in between factors of sex, age, weight, height, playing level and group of instrument. The MANOVA was employed to address the large number of comparisons, and to protect from over- or underestimating the statistical significance. The result of the MANOVA with respect to the chairs revealed that there are substantial differences between the cushioning and the width of the chair´s seat surface. The only significant variables for chair differences were in the group of the pressure data. More specifically, the absolute values for the mean pressure at the left and right side under the thigh and tuber ischiadicum were influenced. Exemplary, Fig 4 shows the results for the mean pressure under the tuber ischiadicum. The results for all five chairs are displayed, and the mean pressure is plotted against the weight of the subjects. The positive correlation between pressure and weight is obvious, as the supporting surface does not increase with the same rate as the weight of the subjects. More interesting is the fact that the two chairs (1 and 4) with the wide, soft cushioning surface exhibit a 25% reduction of the pressure load. Obviously subjects with skin problems due to pressure load should use the wide soft cushioned chairs instead of the chairs with a narrow cushioned or hard surface (such as chair 3 or 5).

The additional weight of the instruments can only explain a small part of the difference between the two conditions (without instrument vs. with instrument). Professional players had a force difference of up to 95N while playing an instrument. As the additional weight of the instrument was already considered, this additional force might be due to a tighter sitting position. This force might be useful in putting more effort into playing the instrument.

In general, older subjects rated the comfort of the chairs worse than younger subjects (Table 4). It is out of the scope of this study if elder subjects really disliked all chairs less, or only used a different intrinsic measure scale. Within this study, the two chairs with softest cushioning, as well as widest seating surface (chair 1 and 4), have the same relationship with regards to age: the older the subject, the more they disliked the chair. This is especially interesting, as within this study elderly people are heavier, and therefore would benefit from the softer cushioning. Two of the chairs with high mean pressure loads had significant correlation with age. However, the correlation was the opposite for the two chairs. While the wooden stool (chair 5) was generally more disliked with increasing age of the subjects, chair number two was liked better the older the subject was. The second chair is the tallest chair, resulting in a knee flexion angle of approximately 120°, and bringing the subjects closer to a standing position. More important than the knee flection is, however, the hip flection. The hip flection influences the lumbar lordosis as an important factor to maintain an ergonomic posture.

Still, the pressure on the seating surface is equivalent to other chairs with the same cushioning. Therefore, the support of the second chair was comparable to the others. As older people seemed to rate this chair better, it could be speculated that older musicians prefer a supported but standing position.

Furthermore, there is no significant difference between the subjective assessment of all chairs in male subjects whilst female subjects rate the ergonomically formed sitting roll best (note 2) and the wooden chair worst (note 5). Since these two chairs have the hardest surface of all chairs, the difference in rating might be due to the ergonomic form. It could be speculated that women pay more attention to the seat surface than to the cushioning level.

With respect to the **second** hypothesis, we have found some relationship between the sitting position and the playing level. The only significant variables were upper body posture variables and more specifically, the scapular width and the scapular height. However, considering the demographic differences between the three playing levels, these differences have to be handled with care. Scapular width was larger for the professional players. However, this group also consisted mainly of older male players with more weight and larger body size. The other two groups had more female musicians. The scapular distance was reduced in these two groups, but not significantly different between them.

Chair number 6 could only be measured with the back scan. The upper body posture variables only showed differences in the scapular distance and scapular height, which we associate with the difference in the population of the two groups. Consequently, no quantitative statement can be given for this chair.

In summary, no relationship between the upper body posture data and the pressure data could be found. More specifically, a simple factor analysis revealed that these two dimensions are more or less independent, at least within sitting on a chair. The cushioning of the chairs reduces the pressure significantly, while the loaded area seems to stay constant. When using an instrument, professional musicians increase the pressure onto the sitting surface.

## Conclusion

The chair cushioning decreases the mean pressure while sitting by about 30%. Playing with an instrument puts an additional force onto the surface of the chair that is more than the weight of the instrument. In the sitting position no relationship between pressure data and upper body posture data could be found. Therefore, it is speculated that the intersubject variability is larger than systematic differences introduced by the chair or instrument.
